# High Temporal Beta‐Diversity of Pollinators in Early Successional Forests After Windthrow

**DOI:** 10.1002/ece3.71571

**Published:** 2025-06-17

**Authors:** Elena Gazzea, Luca Conti, Emanuele Rossi, Pierfilippo Cerretti, Maurizio Mei, Dino Paniccia, Andrea Battisti, Lorenzo Marini

**Affiliations:** ^1^ Department of Agronomy, Food, Natural Resources, Animals and Environment (DAFNAE) University of Padova Legnaro Italy; ^2^ Department of Biology and Biotechnology “Charles Darwin” (BBCD) Sapienza University of Rome Rome Italy; ^3^ Independent Researcher Frosinone Italy

**Keywords:** flower‐visiting insects, natural disturbances, *Picea abies*, secondary succession, species replacement, Vaia storm

## Abstract

Forest windthrows have generally a positive effect on the biodiversity of understory vegetation and on insects associated with open conditions, such as pollinators. However, the early successional stage after forest disturbances can be highly heterogeneous in both space and time. While most of the available literature has focused on finding spatial patterns of biodiversity in relation to the disturbed environment, few studies have measured the temporal changes occurring in wind‐affected forests, especially within the early successional stage. Here, we focused on describing the temporal dynamics of vascular plants and pollinators by sampling the same sites after 3 and 5 years from a major storm event (Vaia) in the Italian Alps. We observed high values of temporal β‐diversity for all pollinator taxa, with species belonging to Diptera, i.e., hoverflies and tachinids, experiencing the highest temporal community change, followed by Hymenoptera (wild bees). Conversely, the temporal change in the understory plant communities was the lowest. For all studied taxa, the temporal dynamics were mostly driven by the turnover of species in the assemblages, indicating that colonising species largely replaced original species. Furthermore, we detected an important component of species gain over time in the pollinator communities, while plant communities experienced little net species change. Although we did not find significant environmental patterns explaining the overall temporal community dissimilarity, we found that mountain topography may drive some components of temporal change, especially for the dipteran communities. Our results indicate that organisms with higher ecological and trophic requirements experience higher species turnover in early successional forests, as these evolve over time. We stress the importance of monitoring arthropod assemblages in disturbed forests and suggest to carefully evaluate single‐year studies for management and conservation purposes.

## Graphical abstract

We described the temporal dynamics of vascular plants and pollinating insects by sampling the same windthrow sites after 3 and 5 years from a major storm event in the Italian Alps. Pollinator communities exhibited high temporal β‐diversity, while the understory plant community changed the least. We observed increasing temporal variation in taxa consistent with the complexity of their trophic interactions and ecological requirements.

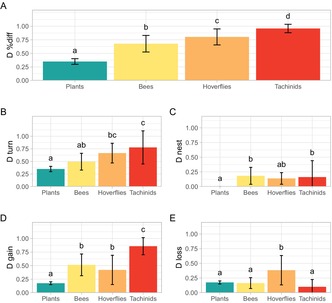

## 1 Introduction

Natural disturbances are important drivers of forest dynamics (Seidl and Turner 2022). By disrupting the prevailing structure of forests and altering soil conditions, canopy cover, and the availability and distribution of resources and microhabitats, disturbances such as windthrows have a significant impact on forest biodiversity (Swanson et al. 2011; Thom and Seidl 2016; Viljur et al. 2022). Although the effects on biodiversity largely depend on the taxonomic group and the post‐disturbance management strategy (Georgiev et al. 2022; Thorn et al. 2018; Wermelinger et al. 2017, 2025), the increase in light conditions and deadwood availability following windthrows has been shown to generally benefit the understory vegetation layer (Cacciatori et al. 2022; Dietz et al. 2020; Marangon et al. 2022) and several arthropod groups (Cours et al. 2023; Duelli et al. 2002), especially those taxa associated with open canopies, such as flower‐visiting insects (Gazzea et al. 2025; Perlík et al. 2023).

Many flower‐visiting insects are important plant pollinators. Their ecology can be very different as pollinators belong to different taxonomic groups. For example, bees (Hymenoptera) rely on nectar and pollen from plants around their nest, while hoverflies (Diptera: Syrphidae) feed on flowering plants as adults, but rely on other substrates as larvae (Rader et al. 2016). Other flies, such as tachinids (Diptera: Tachinidae) are parasitoids during their larval stage, but visit flowers during their adult stage, potentially being pollinators as well (Cerretti 2010). In natural disturbance studies, e.g., Viljur et al. (2022) and Wermelinger et al. (2017), pollinators are often included as a functional group or as part of large orders (e.g., Hymenopterans) in multi‐taxonomic assessments without specific study of them, although the characteristics of the disturbance may drive their diversity differently, depending on their ecology.

While the effects of windthrows on arthropod biodiversity have been often investigated in relation to their spatial heterogeneity (Bouget 2005; Cours et al. 2022; Eckerter et al. 2022; Gazzea et al. 2025; Kortmann et al. 2024), studies have typically been conducted within a single year, thereby capturing only a temporal snapshot along the forest succession. Cumulatively, most studies on windthrows have covered a period of 5 years or less after the storm, so their long‐term effects are rarely documented (Thorn et al. 2018). As exceptions, Duelli et al. (2002), Georgiev et al. (2022), and recently, Wermelinger et al. (2025) found that the changes caused by wind disturbance on the species richness and on the community composition of several arthropod groups persisted for over a decade, after which the similarity relative to intact forests increases as an effect of successional dynamics.

Yet, the diversity of forest‐dwelling organisms can greatly differ not only between but also within successional stages (Hilmers et al. 2018). When assessing the biodiversity change over time, most studies have applied a space‐for‐time substitution (Hilmers et al. 2018; Viljur et al. 2022) and only very few studies in Europe or elsewhere have used permanent plots to control for the successional dynamics of disturbed sites (also reviewed by Wermelinger et al. 2025). Recently, Wermelinger et al. (2025) exceptionally examined the trajectories of species diversity over 20 years in the same plots, finding an initial boost of arthropod species richness and abundance, the latter rapidly declining over time while species numbers remained at relatively high levels. Previously, Duelli et al. (2002) and Wermelinger et al. (2017) observed variable trends in the species richness of several arthropod taxa sampled, respectively, 1 and 10 years, and 2 and 5 years after a severe storm. Yet, the sampling years are deemed too close to reveal temporal trends in the arthropod diversity (Wermelinger et al. 2017) and they are not further examined.

When monitoring biodiversity change, the sole use of species richness as a biodiversity indicator might be inadequate (Hillebrand et al. 2018). The interpretation of trends in temporal biodiversity requires tools that integrate the identity of species and the directionality of change linked to local extinction and immigration patterns (Dornelas et al. 2014). A considerable number of methods have been proposed to assess the compositional change of species communities between two or more sites or over time, i.e., the assemblage β‐diversity (Anderson et al. 2011; Baselga 2010; Legendre 2014). Recently, Legendre (2019) proposed a method for the analysis of temporal changes in community composition through time from repeated surveys at several sites, which has already been applied across different ecosystems (Cooke et al. 2023; Freitag Kramer et al. 2025; Lawson et al. 2024). However, rather than focusing only on reporting the prevalence and direction of change in β‐diversity, it is also important to clarify the underlying mechanisms driving community homogenization or differentiation to enhance our understanding of ecological systems (Rolls et al. 2023).

Here, we used β‐diversity measures to study the temporal dynamics of understory vegetation and pollinator communities within the early successional stage after a large‐scale storm event in the Italian Alps. To do so, we sampled vascular plants, wild bees, hoverflies, and tachinids in permanent windthrow‐affected sites 3 and 5 years after the storm. We aimed to (i) quantify the changes in the communities over a 5‐year time, (ii) test whether the temporal change differed across taxa and (iii) test how the temporal change of the considered communities was related to the environment. Since pollinators comprise taxa with different ecologies, we expect a taxon‐specific temporal community change.

## 2 Materials and Methods

### 2.1 Study Area and Sampling Design

**FIGURE 1:**
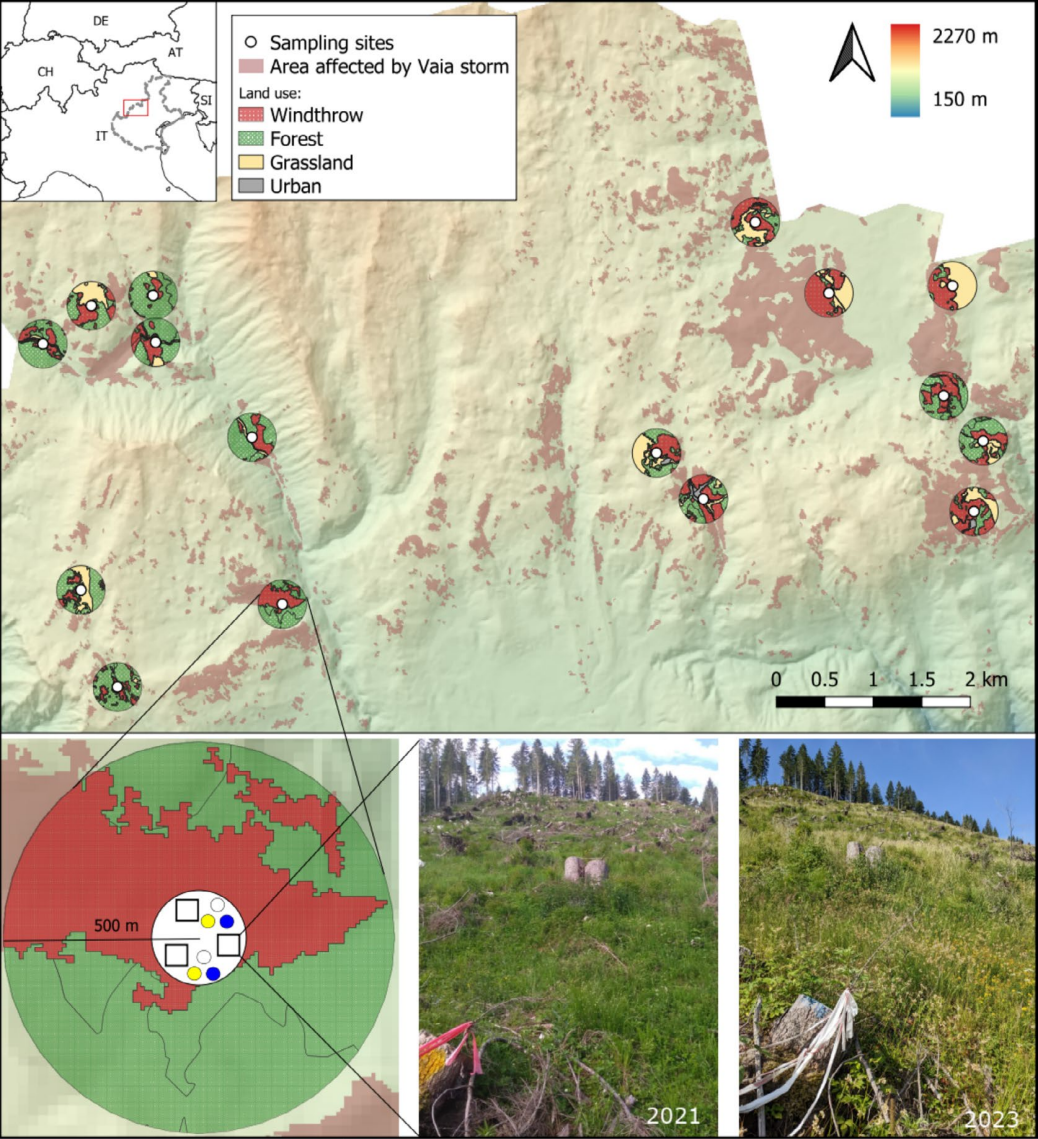
Map of the distribution of the sampling sites (white dots) within the study area and schematic representation of the sampling design. Inset map shows the location of the study area within the Veneto administrative region in Italy. Area damaged by Vaia storm is highlighted in red. Circular buffers indicate the land use in a 500 m radius from the sampling sites. In each sampling site, three vegetation plots (squares) and two triplets of pan traps (yellow, white, and blue circles) were laid out. The sampling was repeated in 2021 and 2023.

This study was conducted in the Asiago Plateau, a mountainous region located in the Venetian Pre‐Alps, in north‐eastern Italy (Figure 1). During 27–30 October 2018, this area was severely hit by the Vaia storm, a strong depression that triggered wind gusts of up to 200 km/h, storm surges, and heavy rains, with more than 700 mm of cumulative precipitation in 72 h (Pellegrini et al. 2021). This extreme event caused massive windthrows in the north‐eastern Italian Alps, with more than 42,500 ha of forests affected and about 8.5 million cubic meters of wood left on the ground (Chirici et al. 2019). Forests damaged by the storm Vaia were pure and mixed stands mainly composed of Norway spruce ( *Picea abies* ) (Marangon et al. 2024). In this study area, we selected 16 sampling sites affected by windthrow and subsequently salvaged logged shortly after the storm and within similar time frames. The selected windthrow sites were the centres of circular buffers of 500 m radius, so that the minimum distance between sites was 1 km (from the centre of a buffer to another). The average nearest neighbour distance between sampling sites was 1.74 km. Sampling sites varied in elevation (min 1144, max 1610 m a.s.l.) and in landscape composition (Table 1). To describe the landscape surrounding the sampling sites we calculated the percentage cover of windthrow gaps, forests, grasslands, and urban areas using the official windthrow data published in the Veneto Region geoportal (Regione del Veneto 2021) and the merged categories from the Corine Land Cover data updated to 2020 in buffers of 500 m radius. We followed the same sampling design as in Gazzea et al. (2025).

**TABLE 1:** Minimum, maximum, and mean (±SD) percentages of forest, grassland, windthrow and urban areas in a landscape of 500 m radius around the sampling sites.

Land use type	Min	Max	Mean ± SD
Forest	7	83	48 ± 24
Grassland	0	53	17 ± 15
Windthrow	14	62	34 ± 13
Urban areas	0	9	1 ± 2

### 2.2 Pollinator Sampling

In each sampling site, we placed two triplets of pan traps c. 60 m apart (16 sites × 6 pan traps = 96 pan traps in total), each triplet consisting of a yellow, a blue, and a white bowl containing c. 250 mL of 75% propylene glycol and a drop of detergent (Droege et al. 2016). Traps were emptied and reset fortnightly, and all captured insects were stored in 70% ethanol. We pooled community data at the site level by merging the captures from the two triplets. We opted for the use of pan traps to catch a greater diversity of species belonging to multiple pollinator taxa, which is not feasible with standard transect counts (O'Connor et al. 2019). We conducted sampling activities in 2021 and in 2023, respectively three and five years after the storm event. In 2021, we repeated the pollinator sampling three times throughout the season, from June to August. In 2023, we repeated the sampling an additional time because hail damaged most of the pan traps of one sampling round. However, the additional sampling round was also damaged by extreme rainfall. To have comparable estimates, we decided to retain two complete sampling rounds for each year, one in June and one in August (1st round in 2021: 17/06–03/07, 2nd round in 2021: 31/07–13/08; first round in 2023: 27/06–10/07, second round in 2023: 07/08–21/08). Captures from different sampling rounds were pooled to obtain yearly estimates. All collected specimens belonging to the superfamily Apoidea: Anthophila (‘bees’) and to the families Syrphidae (‘hoverflies’) and Tachinidae (‘tachinids’) were identified to species by experts, respectively M.M., D.P., and P.C., using several taxonomic resources (Appendix B), voucher specimens from private and public collections, and taxonomic expertise. All specimens are kept in the collections of the DAFNAE department at the University of Padova, Italy, except for some bee duplicates, which are kept at the Museum of Zoology at the University “Sapienza” of Rome, Italy.

### 2.3 Vegetation Sampling

At the beginning of the sampling activity in 2021, we randomly established three 5 × 5 m vegetation plots in each sampling site (16 sites × 3 plots = 48 vegetation plots in total). First, we visually estimated the total plant cover in each plot. Second, we identified all vascular plants in the herbaceous layer to species level. Third, we visually estimated the relative biomass (%) of each plant species, so that the sum of the estimated biomass of all species present was 100 (Ónodi et al. 2017; Redjadj et al. 2012). In 2023, we repeated the vegetation sampling in the same plots established in 2021. Two vegetation plots in two different sampling sites were damaged by grazing in 2023 and were thus excluded from the analyses, i.e., the plant community analysis for two sampling sites is based on two vegetation plots instead of three.

### 2.4 Calculating Community Temporal Changes

To calculate and describe the temporal changes in the species assemblages, we followed an approach similar to that of Marta et al. (2021). Temporal β‐diversity in the vegetation, wild bee, hoverfly, and tachinid community composition was calculated using Odum's percentage difference, *alias* Bray–Curtis dissimilarity index (*D*_%diff_) (Baselga 2013; Legendre 2019) between pairs of temporally distinct assemblages (*t*_1_: year 2021 vs. *t*_2_: year 2023) for each taxonomic group. The index was computed using the Formula (1),

(1) $$\left(D_{\%\text{diff}}=\frac{B+C}{2A+B+C}\right)$$

where *A* designates the sum of the minimum abundances of the species at the two time frames under comparison, i.e., the shared species abundances between 2 years, *B* is the sum of species abundances at t_1_ minus *A*; and *C* is the sum of abundances at t_2_ minus *A* (Legendre 2014). The percentage difference index is the abundance‐based form of Sørensen dissimilarity index described by Baselga (2010), obtained by replacing the frequencies *a*, *b* and *c* in the Sørensen index for presence–absence data by the quantities *A*, *B* and *C* defined above (Legendre 2014). Consequently, the quantity *A* refers to the species that persisted between t_1_ and t_2_, *B* refers to the species that went locally extinct, and *C* refers to the species colonising the site.

The temporal β‐diversity index calculated for each taxonomic group was further partitioned into its additive turnover and nestedness components (Baselga 2010; Legendre 2014). Temporal turnover reflects species replacement over time and was calculated as (2)

(2) $$\left(D_{\text{turn}}=\frac{\min\left(B,C\right)}{A+\min\left(B,C\right)}\right)$$

while the nestedness reflects the tendency of two assemblages to be subsets of one another, with arriving species independent of original species (Baselga 2010, 2012) and was calculated as (3)

(3) $$\left(D_{\text{nest}}=\frac{\left|B-C\right|}{2A+B+C}\times \frac{A}{A+\min\left(B,C\right)}=D_{\%\text{diff}}-D_{\text{turn}}\right)$$

The dissimilarity indices and their turnover and nestedness‐related components were calculated using the *beta.div.comp* function from the *adespatial* package (Dray et al. 2023).

To better understand the effects of local species colonisation and extinction on temporal β‐diversity at each sampling site, we also decomposed the percentage difference index (*D*_%diff_) into additive gain (*D*_gain_) and loss (*D*_loss_) components. Using the same notation as above, *D*_gain_ indicates the abundances‐per‐species gains and is calculated as (4)

(4) $$\left(D_{\text{gain}}=\frac{C}{\left(2A+B+C\right)}\right)$$

and *D*_loss_ reflects the abundances‐per‐species losses and is calculated as (5)

(5) $$\left(D_{\text{loss}}=\frac{B}{\left(2A+B+C\right)}\right)$$

where *D*_gain_ + *D*_loss_ = *D*_%diff_ (Legendre 2019). High temporal β‐diversity values indicate the magnitude of change and can reflect both high losses or high gains. Therefore, to aid interpretation, we also calculated the net temporal change, i.e., gains minus losses, for each community within a site in relation to other sites, and we tested the significance of the mean change across all sites using paired *t*‐tests and 999 permutations (Legendre 2019). Positive change values indicate net gain, and negative values indicate net loss. For each community, we produced B‐C plots to display the relative importance of the loss (B in Equation 5) and gain (C in Equation 4) components across sites through time (Legendre 2019). We calculated the gain and loss components of temporal β‐diversity with the *TBI* function from the *adespatial* package (Dray et al. 2023).

### 2.5 Modelling Community Temporal Changes

We compared the temporal β‐diversity and its components across taxa using linear mixed effects models (*lme4* package, Bates et al. 2015). We used *D*_%diff_, *D*_turn_, *D*_nest_, *D*_gain_, and *D*_loss_ as response variables and taxon as an explanatory variable. We included site ID as a random factor as multiple taxa were sampled in the same sites. Differences in dissimilarity indices among taxa were assessed by post hoc Tukey test. Next, we used linear mixed effects models with a Gaussian error distribution (*lme4* package) to link the temporal changes in community composition to the environment. We used *D*_%diff_, *D*_turn_, and *D*_gain_ as response variables and the interaction between taxon and elevation and between taxon and windthrow extent (% windthrows in the landscape, considering a 500 m radius buffer) as explanatory variables. Site ID was included as a random factor. We calculated conditional *R* ^2^ using the function r.squaredGLMM (*MuMIn* package, Bartoń 2023). Continuous predictors, i.e., elevation and windthrow extent, were tested for collinearity by calculating the Pearson's correlation coefficient using the function cor.test (*stats* package) (*r* = −0.24, *p* = 0.363). We standardised continuous predictors to have zero mean and unit variance (*Z*‐scores). Model residuals were inspected for normality and constant variance using diagnostic plots (Figure S1). To evaluate potential collinearity issues among explanatory variables, we computed the variation inflation factors (VIFs) using the *car* package (Fox and Weisberg 2019) on the model without interactions. We did not detect VIFs > 2. Analyses were run with the Software R version 4.3.1 (R Core Team 2023).

## 3 Results

### 3.1 General Results

**FIGURE 2:**
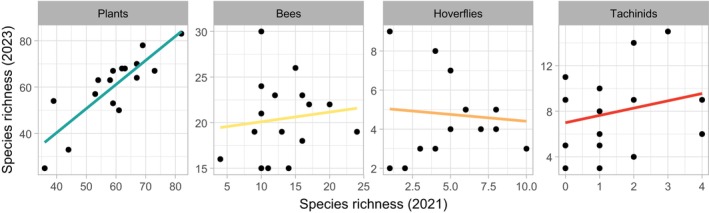
Scatterplot of number of species recorded in the first year of sampling (2021) vs. number of species recorded in the second year of sampling (2023) for each taxon.

After three years from the storm, i.e., in year 2021, we recorded in total 497 wild bees (64 species), 248 hoverflies (25 species), 28 tachinids (16 species), and 223 plant species. After five years from the storm, i.e., in year 2023, we recorded 1096 wild bees (69 species), 290 hoverflies (26 species), 247 tachinids (44 species), and 230 plant species. The number of species recorded in each site did not correlate between sampling years, except for plants (Pearson correlation coefficient of plants: *r* = 0.84, *p* < 0.0001; bees: *r* = 0.12, *p* = 0.66; hoverflies: r = −0.09, *p* = 0.754; tachinids: *r* = 0.24, *p* = 0.366) (Figure 2), indicating that sites rich in pollinator species in the first year of sampling were not necessarily rich in species also in the second year of sampling. We report the full list of recorded species in Table S1 and Table S2.

### 3.2 Community Temporal Changes Across Taxa

**FIGURE 3:**
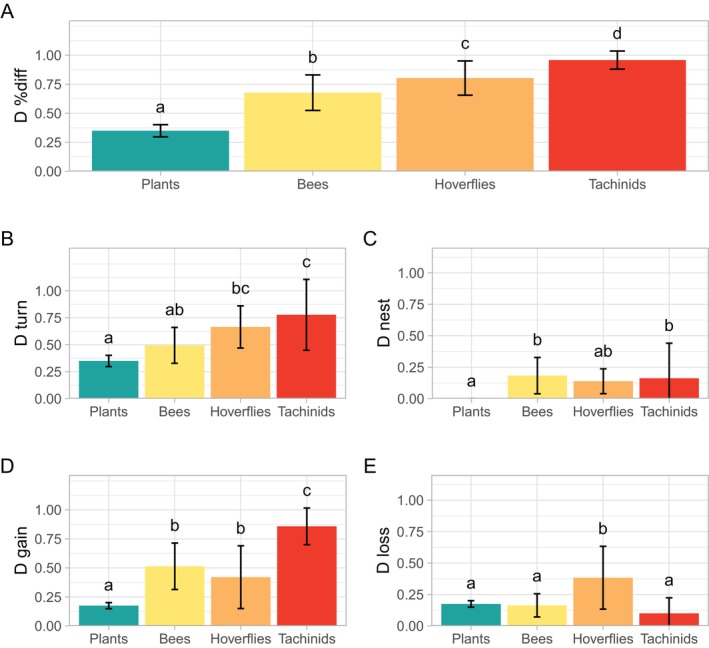
Temporal β‐diversity across taxa sampled in windthrows. For each taxon, we show (A) overall community change (*D*_%diff_) and its components (B) turnover (*D*_turn_) and (C) nestedness (*D*_nest_), and (D) gains (*D*_gain_) and (E) losses (*D*_loss_). All indices were computed from species abundance data. Bar plots show mean and standard deviation values. Letters indicate significant differences in the Tukey post hoc test.

We found different patterns of temporal β‐diversity across taxa (Figure 3). The overall dissimilarity index (*D*_%diff_) was the lowest for plant communities, moderately high for bees and hoverflies, and the highest for tachinids (mean ± SD hereinafter: plants = 0.35 ± 0.05, bees = 0.68 ± 0.15, hoverflies = 0.80 ± 0.15, tachinids = 0.96 ± 0.08) (Figure 3A, Table A1). When partitioning the index into its turnover and nestedness components, we found that most of the change in communities was due to species turnover (*D*_turn_). The temporal species replacement (*D*_turn_) in insect communities was generally high across all taxa (bees = 0.49 ± 0.17, hoverflies = 0.67 ± 0.20, tachinids = 0.78 ± 0.33), while it was lower (0.35 ± 0.05) and represented all the temporal β‐diversity for plants (Figure 3B, Table A1). The nestedness‐related component (*D*_nest_) was low for all studied taxa, indicating that the communities inhabiting windthrows 5 years after the storm were not specific subsets of the communities found previously (Figure 3C, Table A1).

**FIGURE A1:**
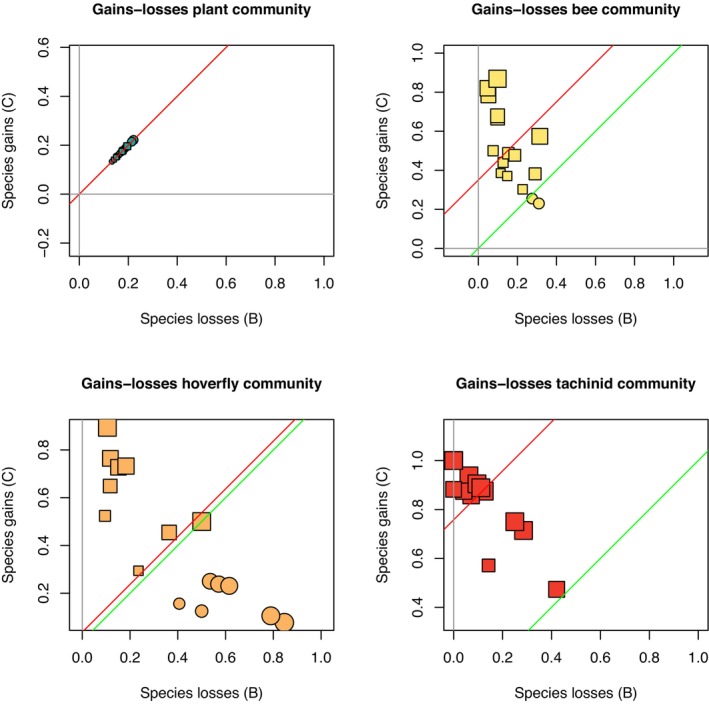
B–C plots for plant, bee, hoverfly and tachinid communities (abundance data) sampled in 16 windthrows. Squares represent sites where gains > losses. Circles represent sites where gains<losses. Size of symbols is proportional to the temporal beta‐diversity values and colour is the same as in Figure 2. Green line with slope of 1 indicates line where gains equal losses. The red lines are drawn parallel to the green line, i.e., with a slope of 1, passing through the centroid of the points. Red lines above the green line indicate that, on average, species gains dominated losses between 2021 and 2023. This is significant (paired *t*‐test with 999 permutations) for bee and tachinid communities, while no significant net gain was observed for plant and hoverfly communities.

The partition of the temporal β‐diversity into gains and losses indicated a higher share of the abundances‐per‐species gains (*D*_gain_) in the communities of pollinators than in plants (plants = 0.17 ± 0.02, bees = 0.51 ± 0.20, hoverflies = 0.42 ± 0.27, tachinids = 0.86 ± 0.16) (Figure 3D, Table A1). The mean of the differences between gains and losses across all sites was significantly positive for bees (results of the paired *t*—test using 999 permutations: mean = 0.35, *p* ≤ 0.05) and for tachinids (mean = 0.76, p ≤ 0.05), indicating net mean gains in these communities over time (Figure A1). Conversely, although the share of gains in the hoverfly communities was high, approximately half of the sites experienced losses (*D*_loss_) (hoverfly = 0.38 ± 0.25), resulting in non‐significant mean gains (Figure 3E, Figure A1). The difference between mean gains and mean losses was minimal for plants (Figure 3E, Figure A1).

### 3.3 Environmental Drivers of Community Temporal Changes

**FIGURE 4:**
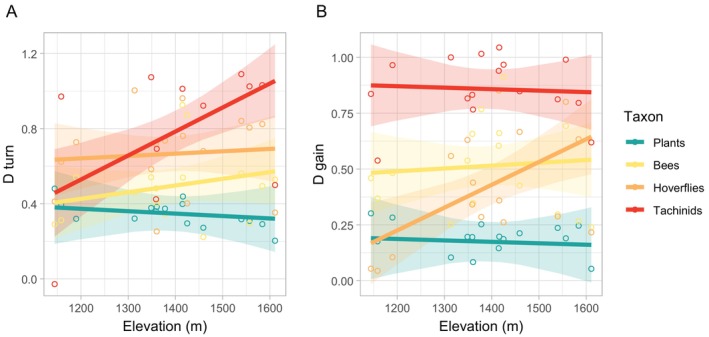
Effect of elevation on the (A) turnover (*D*_turn_) and (B) gain (*D*_gain_) components of temporal β‐diversity on the considered taxa. Raw data points and 95% confidence intervals are plotted alongside linear fit.

We did not detect significant effects of elevation and percentage of windthrows in the landscape on the overall β‐diversity index for any of the studied taxa (*R* ^2^ = 0.82) (Table 2). However, we observed that the taxonomic turnover of tachinids was higher in sites at higher elevations (*R* ^2^ = 0.61) (Table 2; Figure 4). Similarly, the gain component of hoverflies was higher at higher elevations (*R* ^2^ = 0.73) (Table 2; Figure 4).

**TABLE 2:** Summary table displaying estimates (Est), confidence intervals (95% CI, lower and upper boundaries), and standard deviation of the random terms from the linear mixed effect models testing the effects of the interaction between taxon and elevation and between taxon and percentage of windthrows in the landscape on the temporal β‐diversity index and its turnover and gain components.

Fixed effects	*D*_%diff_			*D*_turn_			*D*_gain_		
	Est	CI (lower)	CI (upper)	Est	CI (lower)	CI (upper)	Est	CI (lower)	CI (upper)
Intercept	0.349	0.289	0.409	0.349	0.256	0.441	0.174	0.087	0.262
Bees	0.329	0.251	0.407	0.146	0.029	0.263	0.339	0.223	0.456
Hoverflies	0.455	0.376	0.533	0.316	0.199	0.434	0.246	0.129	0.362
Tachinids	0.610	0.532	0.688	0.427	0.295	0.560	0.684	0.567	0.800
Elevation	−0.018	−0.080	0.044	−0.018	−0.114	0.078	−0.009	−0.100	0.082
% windthrow	0.003	−0.060	0.065	0.003	−0.094	0.099	0.001	−0.090	0.093
Bees: elevation	0.050	−0.031	0.132	0.068	−0.054	0.190	0.027	−0.094	0.148
Hoverflies: elevation	0.004	−0.078	0.085	0.036	−0.086	0.157	0.152	0.031	0.273
Tachinids: elevation	0.022	−0.059	0.103	0.197	0.064	0.329	0.000	−0.121	0.121
Bees: %windthrow	0.047	−0.035	0.128	0.014	−0.107	0.136	0.076	−0.045	0.197
Hoverflies: %windthrow	−0.022	−0.104	0.059	−0.032	−0.154	0.090	0.097	−0.024	0.218
Tachinids: %windthrow	0.022	−0.059	0.104	0.134	0.002	0.267	0.040	−0.081	0.161

Random effects	SD	SD	SD
Site ID	0.048	0.085	0.064
Residual	0.110	0.163	0.163

## 4 Discussion

Our observational study focused on describing the temporal dynamics of vascular plants and pollinating insects within the early successional stage of a forest recovering from a large windthrow disturbance. Between 3 and 5 years from the storm, pollinator communities exhibited high temporal β‐diversity, while the understory plant community changed the least. The temporal dynamics of pollinators were mostly driven by the turnover and by the gain of species in the assemblages 5 years after the storm, while plant communities experienced little net species change. Mountain topography drove some components of temporal change, especially for the dipteran communities, but the overall temporal community dissimilarity remained strongly linked to the rapid evolution of forest succession and to the specific taxon response. In particular, we observed increasing temporal variation in taxa consistent with the complexity of their trophic interactions.

The patterns in the temporal β‐diversity found in the plant communities between 3 and 5 years from the disturbance reflected the general patterns of vegetation development in the early stage of succession in storm‐affected coniferous forests (Lain et al. 2008). Previous research indicates that the loss of tree cover determined by wind disturbances increased the ground layer cover by nearly double and the arrival of 35%–50% new pioneer species immediately after the disturbance, i.e., within the first 2 years (Lain et al. 2008). However, 4 years after the storm, Lain et al. (2008) reported little changes in the ground layer vegetation of salvaged plots, with the total cover remaining very high. In particular, some bryophyte species declined, while the cover of ruderal sedges (*Carex* spp.) and shrubs, such as raspberry ( *Rubus idaeus* ) and blueberry (*Vaccinium* sp.) continued to increase. These results indicate that there is a high turnover of species in the understory shortly after the disturbance, after which the proportion of early colonisers decreases with time following the disturbance and the plant community evolves slower towards being shrub‐ and tree‐dominated (Ulanova 2000). The topography of mountain regions influences the long‐term successional dynamics of understory vegetation, with a lower regeneration speed at higher elevations (Marangon et al. 2024). Consistently, we observed a little decrease in plant species turnover with elevation, although the effect over the short time period we considered was not significant.

Over a decade after a major storm event, Georgiev et al. (2022) found little within‐windthrow β‐diversity (called β‐dispersion) for vascular plants and bees and wasps, suggesting that windthrows lead to homogeneous communities in the mid‐ to long‐term. However, the β‐dispersion was measured in 1 year, so the results represent community homogenisation, not biotic homogenisation over time (Olden and Rooney 2006). The high values of temporal β‐diversity we observed for the pollinator communities reflected strong compositional changes, thus indicating low compositional stability over time. We observed large between‐taxa variability for pollinators that could be in part explained by the mobility and resource needs of the different groups (Rader et al. 2016; Stireman et al. 2006). As previously reviewed, disturbances have high temporal heterogeneity and their environmental effects on biotic communities can be transient, long‐lasting, immediate, delayed, or cumulative over time, depending on the taxon (Cours et al. 2023). Tachinids were those with the largest beta‐diversity, followed by hoverflies and wild bees. Most wild bees are central‐place foragers, i.e., they require nectar and pollen within foraging distance of their nest site and, although mobile organisms, they are strictly linked to their nesting habitat (Kendall et al. 2022). Wild bees are also primary consumers, as they feed on nectar and pollen from plants present in the site. Hence, the wild bee community found in disturbed habitats is linked to some extent to the abiotic and biotic conditions established in the previous years, and new species are likely to colonise the disturbed sites with time. Similar to wild bees, hoverflies depend on flowers for nectar as adults, but they are not central foragers, exploring the landscapes at a larger spatial scale (Lami et al. 2021). Hoverflies also require different resources for different stages of their life cycle, e.g., their larvae require different microhabitats to develop, being predatory of other organisms or saprotrophic. The community of hoverflies may be thus less bound to the characteristics of the windthrows by depending on additional resources. Finally, tachinids are parasitoids, i.e., free‐living pollinators as adults but endophages during their larval stage, meaning that their larvae develop inside a host organism, usually other primary consumer arthropods. The observed higher temporal change in tachinids, with a significant increase in species richness and abundance, was likely driven by the availability of suitable host species for their larvae more than by local or landscape features. As windthrows likely impacted host availability for tachinids and altered microhabitats for hoverfly larvae, the dipteran community likely experienced indirect and delayed effects from the storm, leading to possible long‐term shifts in community composition as resources recover during the successional development.

In the early successional stage, windthrows may represent hotspots for the opportunistic foraging and development of insects from the surrounding habitats, regardless of within‐windthrow local and landscape conditions (Gazzea et al. 2025). Pollinator species may, in the short term, exploit the resources created by large‐scale storm events without consistent relationships with the environment. Here, we observed that there is also a high temporal variation, and that this is higher with increasing trophic complexity and greater ecological requirements. It appears that not only different functional groups of arthropods have different trajectories of species numbers and abundances and show a distinct succession (Wermelinger et al. 2025) but also different taxonomic groups within the same functional group. Due to the observed temporal instability in the composition of pollinator communities, results from snapshot studies analysing patterns of flower‐visiting insect diversity in disturbed forests (e.g., thrips Masarovič et al. 2022) should be interpreted with caution. However, the scientific literature on the temporal biodiversity change after forest windthrows is extremely scant (Wermelinger et al. 2025). Existing monitoring programs tend to focus on single assessments over a large spatial scale or frequent assessments on few spatial locations.

Our study shows how repeated observations on the same disturbed areas over time provide a way to track the evolution of the pollinator communities associated with wind‐affected mountain coniferous forests, revealing underlying temporal dynamics that may otherwise be obscured by single‐year observations or aggregated observations across multiple years. It is important to note, however, that the temporal community changes observed in our study cannot be directly linked to the effect of the disturbance event due to a lack of baseline community data. Previous studies comparing communities in windthrows versus intact forests use space‐for‐time substitutions (Gazzea et al. 2025; Wermelinger et al. 2025), as ecological monitoring of pollinator biodiversity in spruce forests is still limited, and the design of before‐after‐control‐treatment experiments are extremely difficult due to the unpredictability of large‐scale storm events. Overall, our study highlights that communities in disturbed environments highly vary over time and tests new indices to detect these temporal changes. The empirical evidence for high compositional turnover and the theoretical indication of the importance of spatial dynamics for this temporal turnover advocate for monitoring programmes over time and space (Hillebrand et al. 2018). Long‐term monitoring further increases the prospects for observing robust effects for large, infrequent disturbances (Turner et al. 2003). Ultimately, ongoing ecological research on windthrows could establish valuable baselines for future studies.

## Author Contributions

**Elena Gazzea:** conceptualization (equal), data curation (lead), formal analysis (lead), investigation (equal), methodology (equal), visualization (lead), writing – original draft (lead), writing – review and editing (lead). **Luca Conti:** investigation (equal), writing – review and editing (equal). **Emanuele Rossi:** formal analysis (supporting), resources (equal), writing – review and editing (equal). **Pierfilippo Cerretti:** resources (equal), writing – review and editing (equal). **Maurizio Mei:** resources (equal), writing – review and editing (equal). **Dino Paniccia:** resources (equal), writing – review and editing (equal). **Andrea Battisti:** funding acquisition (lead), writing – review and editing (equal). **Lorenzo Marini:** conceptualization (equal), methodology (equal), supervision (lead), writing – review and editing (equal).

## Conflicts of Interest

The authors declare no conflicts of interest.

## Supporting information


Data S1.

## Data Availability

The data and code that support the findings of this study are available in Figshare at https://doi.org/10.6084/m9.figshare.27629004.v1.

## Appendix A

**TABLE A1:** Tukey post hoc test results for pairwise comparisons of temporal β‐diversity among taxa.

Temporal β‐diversity	Contrasts	Estimate	SE	df	*t* ratio	*p*‐value
*D*_%diff_	Plants—bees	−0.329	0.038	45	−8.654	< 0.0001
	Plants—hoverflies	−0.455	0.038	45	−11.967	< 0.0001
	Plants—tachinids	−0.610	0.038	45	−16.046	< 0.0001
	Bees—hoverflies	−0.126	0.038	45	−3.312	0.001
	Bees—tachinids	−0.281	0.038	45	−7.392	< 0.0001
	Hoverflies—tachinids	−0.155	0.038	45	−4.080	0.001
*D*_turn_	Plants—bees	−0.146	0.062	40.1	−2.360	0.102
	Plants—hoverflies	−0.316	0.062	40.1	−5.123	< 0.0001
	Plants—tachinids	−0.437	0.07	42.5	−6.274	< 0.0001
	Bees—hoverflies	−0.171	0.062	40.1	−2.763	0.0412
	Bees—tachinids	−0.291	0.07	42.5	−4.181	0.0008
	Hoverflies—tachinids	−0.121	0.07	42.5	−1.732	0.32
*D*_nest_	Plants—bees	−0.183	0.05	40.1	−3.691	0.004
	Plants—hoverflies	−0.138	0.05	40.1	−2.790	0.037
	Plants—tachinids	−0.160	0.056	43.1	−2.870	0.031
	Bees—hoverflies	0.045	0.05	40.1	0.901	0.804
	Bees—tachinids	0.023	0.056	43.1	0.412	0.976
	Hoverflies—tachinids	−0.022	0.056	43.1	−0.389	0.98
*D*_gain_	Plants—bees	−0.340	0.06	45	−5.16	< 0.0001
	Plants—hoverflies	−0.246	0.06	45	−4.069	0.001
	Plants—tachinids	−0.684	0.06	45	−11.318	< 0.0001
	Bees—hoverflies	0.094	0.06	45	1.551	0.417
	Bees—tachinids	−0.344	0.06	45	−5.698	< 0.0001
	Hoverflies—tachinids	−0.438	0.06	45	−7.294	< 0.0001
*D*_loss_	Plants—bees	0.011	0.05	45	0.211	0.997
	Plants—hoverflies	−0.209	0.05	45	−4.116	0.008
	Plants—tachinids	0.074	0.05	45	1.474	0.461
	Bees—hoverflies	−0.220	0.05	45	−4.377	0.004
	Bees—tachinids	0.063	0.05	45	1.263	0.591
	Hoverflies—tachinids	0.283	0.05	45	5.64	< 0.0001

## Appendix B

Taxonomic resources used for identifying insect specimens.

**Bees**

Amiet, F., Herrmann, M., Muller, A., and Neumeyer, R. 2001. Fauna Helvetica 6. Apidae 3: Halictus, Lasioglossum. Centre suisse de cartographie de la faune, Schweizerische Entomologische Gesellschaft, 209 pp.

Amiet, F., Herrmann, M., Muller, A., and Neumeyer, R. 2004. Fauna Helvetica 9. Apidae 4: Anthidium, Chelostoma, Coelioxys, Dioxys, Heriades, Lithurgus, Megachile, Osmia, Stelis. Centre suisse de cartographie de la faune, Schweizerische Entomologische Gesellschaft, 275 pp.

Amiet, F., Herrmann, M., Muller, A., and Neumeyer, R. 2010. Fauna Helvetica 26. Apidae 6: Andrena, Melitturga, Panurginus, Panurgus. Centre suisse de cartographie de la faune, Schweizerische Entomologische Gesellschaft, 317 pp.

Amiet, F., Muller, A., and Neumeyer, R. 2014 Fauna Helvetica 4. Apidae 2: Colletes, Dufourea, Hylaeus, Nomia, Nomioides, Rophitoides, Rophites, Sphecodes, Sistropha. Centre suisse de cartographie de la faune, Schweizerische Entomologische Gesellschaft, 239 pp.

Atlas Hymenoptera (2024) http://www.atlashymenoptera.net/.

Bogusch, P. and Straka, J. 2012. Review and Identification of the Cuckoo Bees of Central Europe (Hymenoptera: Halictidae: Sphecodes). *Zootaxa* 3311: 1–41.

Cappellari, A., Mei, M., Lopresti, M., and Cerretti, P. 2018. BumbleKey: An Interactive Key for the Identification of Bumblebees of Italy and Corsica (Hymenoptera, Apidae). ZooKeys 784: 127–138.

Comba, M. (2015) Hymenoptera: Apoidea: Anthophila of Italy. Catalogue with notes on taxonomy and distribution. https://digilander.libero.it/mario.comba/index.html.

Dathe, H. H. 1980. Die arten der gattung Hylaeus F. in Europa (Hymenoptera: Apoidea, Colletidae). Mitteilungen aus dem zoologischen museum in Berlin 56: 207–294.

Dathe, H.H., Scheuchl, E., and Ockermüller, E. 2016. Illustrierte bestimmungstabelle für die arten der gattung Hylaeus F (manskenbienen) in Deutschland, Österreich und der Schweiz. Entomologica Austriaca supplement: 1–51.

Ebmer, A. W. 1969. Die Bienen des Genus Halictus Latr. s. l. im Großraum von Linz (Hymenoptera, Apidae). Teil I. Naturkundliches Jahrbuch der Stadt Linz: 133–183.

Ebmer, A. W. 1970. Die bienen des genus Halictus Latr. s. l. im Großraum von Linz (Hymenoptera, Apidae). Teil II. Naturkundliches Jahrbuch der Stadt Linz: 19–82.

Ebmer, A. W. 1971. Die bienen des genus Halictus Latr. s. l. im Großraum von Linz (Hymenoptera, Apidae). Teil III. Naturkundliches Jahrbuch der Stadt Linz: 63–156.

Ebmer, A. W. 1987. Die europaischen arten der gattungen Halictus Latreille 1804 und Lasioglossum Curtis 1833 mit illustrierten bestimmungstabellen (Insecta: Hymenoptera: Apoidea: Halictidae: Halictinae). 1.Allgemeiner teil, tabelle der gattungen. Senckenbergiana biologika 68: 59–148.

Ebmer, A. W. 1988. Die europäischen arten der gattungen Halictus Latreille 1804 und Lasioglossum Curtis 1833 mit illustrierten Bestimmungstabellen (Insecta: Hymenoptera: Apoidea: Halictidae: Halictinae). 2. Die untergattung Seladonia Robertson 1918. *Senckenbergiana biologika* 68: 323–375.

Ebmer, A. W. 1988. Kritische liste der nicht‐parasitischen Halictidae Österreichs mit Berücksichtigung aller mitteleuropäischen Arten (Insecta: Hymenoptera: Apoidea: Halictidae). *Linzer biologische Beiträge* 20: 527–711.

Gusenleitner, F. and Schwarz, M. 2002. Weltweite Checkliste der Bienengattung Andrena mit Bemerkungen und Ergänzungen zu paläarktischen Arten (Hymenoptera, Apidae, Andreninae, Andrena). *Entomofauna, Supplement* 10: 1–1280.

Kasparek, M. 2019. Bees in the Genus Rhodanthidium: A Review and Identification Guide. *Entomofauna*, suppl. 24: 1–132.

Michener, C. D. 2007. The Bees of the World. 2nd ed. The Johns Hopkins University Press. Baltimore, 953 pp.

Müller, A. 2015. Palaearctic Chelostoma bees of the subgenus Gyrodromella (Megachilidae, Osmiini): biology, taxonomy, and key to species. *Zootaxa* 3936 (3): 408–420. https://doi.org/10.11646/zootaxa.3936.3.6.

Müller, A. 2018. Palaearctic Osmia bees of the subgenus Hoplosmia (Megachilidae, Osmiini): Biology, Taxonomy and Key to Species. *Zootaxa* 4415, no. 2: 297–329. https://doi.org/10.11646/zootaxa.4415.2.4.

Ortiz‐Sánchez FJ, Jiménez‐Rodríguez, A. J. 1991. Actualización del catálogo de las especies españolas de Anthophorini (Hymenoptera, Anthophoridae). *Boletín de la asociación española de entomología* 15: 297–315.

Pauly, A. 2009 Les espèces de Seladonia Robertson en France et en Europe. Clé provisoire pour l'identification des espèces de Seladonia d'Europe. Document de travail de Atlas—Hymenoptera, version 17 mai 2009. www.atlashymenoptera.net/biblio/Seladonia_Europe.pdf.

Pauly, A., Noël, G., Sonet, G., Notton, D.G., Boevé, J.‐L. 2019. Integrative taxonomy resuscitates two species in the *Lasioglossum villosulum* complex (Kirby, 1802) (Hymenoptera: Apoidea: Halictidae). *European Journal of Taxonomy* 541: 1–43 https://doi.org/10.5852/ejt.2019.541

Praz, C. J. 2017. Subgeneric Classification and Biology of the Leafcutter and Dauber Bees (genus Megachile Latreille) of the Western Palearctic (Hymenoptera, Apoidea, Megachilidae). *Journal of Hymenoptera Research* 55: 1–54.

Straka, J. and Bogusch, P. 2011. Contribution to the Taxonomy of the *Hylaeus gibbus* Species Group in Europe (Hymenoptera, Apoidea and Colletidae). *Zootaxa* 2932: 51–67. https://www.researchgate.net/publication/231211904.

Terzo, M., Iserbyt, S., and Rasmont, P. 2007. Révision des Xylocopinae (Hymenoptera: Apidae) de France et de Belgique. *Annales de la société entomologique de France*, 43, no. 4, 445–491.

Warncke, K. 1992. Die westpaläarktischen Arten der Bienengattung Sphecodes. Bericht der naturforschenden gesellschaft Augsburg 52 (195): 9–64.

**Hoverflies**

Bartsch, H., Binkiewicz, E., Rådén, A., and Nasibov E. 2009a. Blomflugor: Syrphidae. Nationalnyckeln till Sveriges flora och fauna, DH 53a. Artdatabanken, SLU, Uppsala, 406 pp.

Bartsch, H., Binkiewicz, E., Klintbjer, A., Rådén, A., and Nasibov, E. 2009b. Blomflugor: Eristalinae & Microdontinae. Nationalnyckeln till Sveriges flora och fauna, DH 53b. Artdatabanken, SLU, Uppsala, 478 pp.

Claussen, C. and Van De Weyer, G. 2004. A New Species of the Genus Cheilosia Meigen (Diptera, Syrphidae) from the Southern Alps. *Volucella* 7: 61–74.

Doczkal, D., Stuke, J.‐H., and Goeldlin De Tiefenau P. 2002. The Species of the *Platycheirus scutatus* (Meigen) Complex in Central Europe, with description of *Platycheirus speighti* spec. nov. from Alps (Diptera, Syrphidae). *Volucella* 6: 23–40.

Francuski, L. J., Vujić, A., Kovačević, A., Ludoški, J. and Milankov, V. 2009. Identification of the Species of the *Cheilosia variabilis* group (Diptera, Syrphidae) from the Balkan Peninsula Using Wing Geometric Morphometrics, with the Revision Of The Status of *C. melanoparedi* Vujić, 1996. *Contributions to Zoology* 78, no. 3: 129–140.

Goeldlin De Tiefenau, P. 1976. Revision du genre Paragus (Dipt., Syrphidae) de la région paléarctique occidentale. *Bulletin Societé Entomologique de Suisse*, 49: 79–108.

Goeldlin De Tiefenau, P. and Lucas, J. A. W. 1981. Paragus (Dipt., Syrphidae) de Corse et de Sardaigne. *Bulletin Société Entomologique Suisse*, 54: 389–397.

Haarto A. 2014. Identification of the Finnish species of Paragus Latreille, 1804 subgenus Pandasyopthalmus Stuckenberg, 1954 (Diptera, Syrphidae). *Sahlbergia*, 20.2: 2–5.

Hurkmans W. 1993. A Monograph of Merodon (Diptera: Syrphidae). Pt. 1. Tijdschrift voor. *Entomologie*, 136: 147–234.

Ståhls, G., Vujić, A. and Milankov, V. 2008. *Cheilosia vernalis* (Diptera, Syrphidae) Complex: Molecular and Morphological Variability. *Annales Zoologici Fennici* 45: 149–159.

Stubbs, A. E. and Falk, S. J. 2002. British Hoverflies: An Illustrated Identification Guide. 2nd edition. British Entomological and Natural History Society, London, 469 pp.

Van Veen, M. P. 2004. Hoverflies of Northwest Europe: Identification Keys to the Syrphidae. KNNV Publishing, Utrecht, 254 pp.

Vujić, A. and Claussen, C. 1994. *Cheilosia bracusi*, A New Hoverfly from the Mountains of Central and Southern Europe (Diptera: Syrphidae). *Bonner zoologische Beiträge*, 45 (2): 137–146.

Vujić, A., Radenković, S., Likov, L, Trifunov, S. and Nikolić, T. 2013a. Three New Species of the *Merodon nigritarsis* group (Diptera: Syrphidae) from the Middle East. Zootaxa 3640, no. 3: 442–464.

Vujić, A., Radenković, S., Trifunov, S., and Nikolić, T. 2013b. Key for European Species of the Cheilosia proxima group (Diptera, Syrphidae) with a Description of a New Species. *Zookeys*, 269: 33–50.

Vujić, A. and Simič, S. 1999. Genus Eumerus Meigen, 1822 (Diptera: Syrphidae) in Area of Former Jugoslavia. Glasn. *Prirodnih Muzej u Beogradu*, B 40–50 (1995–1998): 173–190.

**Tachinids**

Cerretti, P. 2010. I tachinidi della fauna italiana (Diptera Tachinidae), con chiave interattiva dei generi ovest‐paleartici. Volume I, 573 pp., Volume II, 339 pp. + CD‐ROM. Cierre Edizioni, Verona.

Cerretti, P., Tschorsnig, H.‐P., Lopresti, M., and Di Giovanni F. 2012. MOSCHweb—An Interactive Key to the Genera of the Palearctic Tachinidae (Insecta: Diptera). ZooKeys, 205: 5–18.
