# Supplementary material for: High Temporal Beta‐Diversity of Pollinators in Early Successional Forests After Windthrow
Source: Ecol Evol. 2025 Jun 17;15(6):e71571. doi: 10.1002/ece3.71571 (PMC12171635; doi:10.1002/ece3.71571)
Supplement: Supplementary file 1 — Data S1. [file ECE3-15-e71571-s001.docx]

**Supplementary Information for**

**High temporal beta-diversity of** **pollinators in early successional forests after windthrow**

Gazzea Elena^1^*, Conti Luca^1^, Rossi Emanuele^2^, Cerretti Pierfilippo^2^, Mei Maurizio^2^, Paniccia Dino^3^, Battisti Andrea^1^, Marini Lorenzo^1^

^1^University of Padova – Department of Agronomy, Food, Natural resources, Animals and Environment (DAFNAE), 35020 Legnaro, Italy

^2^Sapienza University of Rome – Department of Biology and Biotechnology “Charles Darwin” (BBCD), 00185 Rome, Italy

^3^Via Colle 13, 03100 Frosinone, Italy (Independent Researcher)

*Corresponding author

Email: elena.gazzea@unipd.it

**Table S1** - List of wild bee, hoverfly, and tachinid species and individuals sampled in all sites in 2021 and in 2023.

| **Group** | **Species** | **Individuals** | |
| --- | --- | --- | --- |
|  |  | **2021** | **2023** |
| Wild bees | *Andrena bicolor* | 0 | 32 |
|  | *Andrena pusilla* | 0 | 19 |
|  | *Andrena* sp.1 | 2 | 0 |
|  | *Andrena* sp.10 | 0 | 1 |
|  | *Andrena* sp.11 | 1 | 0 |
|  | *Andrena* sp.12 | 1 | 0 |
|  | *Andrena* sp.14 | 1 | 0 |
|  | *Andrena* sp.16 | 2 | 0 |
|  | *Andrena* sp.17 | 1 | 0 |
|  | *Andrena* sp.18 | 1 | 0 |
|  | *Andrena* sp.19 | 1 | 0 |
|  | *Andrena* sp.2 | 1 | 3 |
|  | *Andrena* sp.22 | 2 | 0 |
|  | *Andrena* sp.24 | 1 | 0 |
|  | *Andrena* sp.5 | 1 | 4 |
|  | *Andrena* sp.6 | 0 | 1 |
|  | *Andrena* sp.7 | 0 | 1 |
|  | *Andrena* sp.8 | 0 | 1 |
|  | *Andrena* sp.9 | 0 | 1 |
|  | *Anthophora furcata* | 6 | 0 |
|  | *Bombus barbutellus* | 0 | 1 |
|  | *Bombus bohemicus* | 1 | 3 |
|  | *Bombus campestris* | 0 | 2 |
|  | *Bombus gerstaeckeri* | 1 | 1 |
|  | *Bombus hortorum* | 16 | 27 |
|  | *Bombus humilis* | 2 | 7 |
|  | *Bombus hypnorum* | 1 | 6 |
|  | *Bombus lapidarius* | 21 | 24 |
|  | *Bombus mesomelas* | 5 | 3 |
|  | *Bombus pascuorum* | 39 | 167 |
|  | *Bombus pratorum* | 18 | 46 |
|  | *Bombus quadricolor* | 0 | 1 |
|  | *Bombus ruderarius* | 0 | 8 |
|  | *Bombus soroeensis* | 59 | 105 |
|  | *Bombus sylvarum* | 4 | 11 |
|  | *Bombus sylvestris* | 0 | 3 |
|  | *Bombus terrestris/lucorum* | 26 | 35 |
|  | *Bombus wurflenii* | 5 | 8 |
|  | *Chelostoma florisomne* | 8 | 6 |
|  | *Chelostoma rapunculi* | 1 | 0 |
|  | *Coelioxys elongatus* | 0 | 1 |
|  | *Dufourea alpina* | 1 | 0 |
|  | *Dufourea dentiventris* | 2 | 2 |
|  | *Halictus carinthiacus* | 0 | 5 |
|  | *Halictus rubicundus* | 12 | 12 |
|  | *Halictus sexcinctus* | 0 | 1 |
|  | *Heriades truncorum* | 1 | 7 |
|  | *Hoplitis claviventris* | 0 | 12 |
|  | *Hoplitis mitis* | 1 | 0 |
|  | *Hoplitis ravouxi* | 0 | 1 |
|  | *Hoplitis tuberculata* | 2 | 0 |
|  | *Hoplitis villosa* | 1 | 19 |
|  | *Hylaeus alpinus* | 1 | 9 |
|  | *Hylaeus confusus* | 106 | 73 |
|  | *Hylaeus hyalinatus* | 0 | 9 |
|  | *Hylaeus incongruus* | 0 | 5 |
|  | *Lasioglossum aeratum* | 9 | 41 |
|  | *Lasioglossum albipes* | 17 | 20 |
|  | *Lasioglossum bavaricum/cupromicans* | 5 | 25 |
|  | *Lasioglossum calceatum* | 2 | 1 |
|  | *Lasioglossum* cf. *fratellum* | 1 | 0 |
|  | *Lasioglossum fulvicorne* | 2 | 4 |
|  | *Lasioglossum laticeps* | 0 | 2 |
|  | *Lasioglossum medinai* | 7 | 0 |
|  | *Lasioglossum morio* | 5 | 0 |
|  | *Lasioglossum parvulum* | 2 | 1 |
|  | *Lasioglossum pygmaeum* | 0 | 3 |
|  | *Lasioglossum rufitarse* | 44 | 105 |
|  | *Lasioglossum semilucens* | 6 | 4 |
|  | *Lasioglossum villosulum* | 1 | 4 |
|  | *Lasioglossum zonulum* | 4 | 3 |
|  | *Megachile alpicola* | 0 | 26 |
|  | *Megachile analis* | 2 | 1 |
|  | *Megachile centuncularis* | 14 | 0 |
|  | *Megachile lagopoda* | 1 | 0 |
|  | *Megachile ligniseca* | 4 | 5 |
|  | *Megachile nigriventris* | 0 | 2 |
|  | *Megachile pyrenaea* | 1 | 0 |
|  | *Megachile versicolor* | 0 | 6 |
|  | *Megachile willughbiella* | 2 | 0 |
|  | *Nomada* sp. | 0 | 1 |
|  | *Nomada* sp.1 | 1 | 0 |
|  | *Nomada* sp.3 | 3 | 0 |
|  | *Osmia caerulescens* | 0 | 1 |
|  | *Osmia claviventris* | 1 | 0 |
|  | *Osmia gallarum* | 3 | 7 |
|  | *Osmia parietina* | 1 | 4 |
|  | *Osmia* sp. | 1 | 0 |
|  | *Osmia xantomelana* | 0 | 3 |
|  | *Panurgus banksianus* | 1 | 1 |
|  | *Seladonia tumulorum* | 0 | 5 |
|  | *Sphecodes ephippius* | 0 | 1 |
|  | *Sphecodes miniatus/geoffrellus* | 2 | 135 |
|  | *Sphecodes puncticeps* | 0 | 1 |
|  | *Stelis ornata* | 0 | 1 |
| Hoverflies | *Blera fallax* | 2 | 1 |
|  | *Brachypalpoides lentus* | 1 | 1 |
|  | *Cheilosia antiqua* | 0 | 1 |
|  | *Cheilosia canicularis* | 2 | 1 |
|  | *Cheilosia himantopus* | 0 | 1 |
|  | *Cheilosia vulpina* | 1 | 0 |
|  | *Chrysotoxum octomaculatum* | 1 | 0 |
|  | *Chrysotoxum verralli* | 0 | 1 |
|  | *Eristalinus aeneus* | 23 | 19 |
|  | *Eristalis arbustorum* | 1 | 0 |
|  | *Eristalis similis* | 5 | 0 |
|  | *Eristalis tenax* | 18 | 11 |
|  | *Eupeodes corollae* | 2 | 7 |
|  | *Eupeodes luniger* | 2 | 0 |
|  | *Lapposyrphus lapponicus* | 0 | 2 |
|  | *Melanostoma mellinum* | 2 | 1 |
|  | *Melanostoma scalare* | 1 | 0 |
|  | *Merodon cinereus* | 188 | 24 |
|  | *Merodon constans* | 1 | 0 |
|  | *Merodon equestris* | 1 | 0 |
|  | *Myathropa florea* | 0 | 1 |
|  | *Orthonevra nobilis* | 1 | 1 |
|  | *Platycheirus manicatus* | 1 | 0 |
|  | *Platycheirus scutatus* | 1 | 0 |
|  | *Scaeva selenitica* | 1 | 0 |
|  | *Sphaerophoria scripta* | 11 | 9 |
|  | *Sphaerophoria taeniata* | 0 | 5 |
|  | *Sphaerophoria virgata* | 0 | 1 |
|  | *Syritta pipiens* | 0 | 2 |
|  | *Syrphus vitripennis* | 1 | 0 |
|  | *Volucella bombylans* | 3 | 3 |
|  | *Volucella pellucens* | 0 | 1 |
|  | *Xylota abiens* | 4 | 14 |
|  | *Xylota caeruleiventris* | 0 | 2 |
|  | *Xylota ignava* | 0 | 7 |
|  | *Xylota jakutorum* | 0 | 9 |
|  | *Xylota segnis* | 15 | 123 |
|  | *Xylota triangularis* | 1 | 0 |
| Tachinids | *Acemya acuticornis* | 0 | 1 |
|  | *Belida angelicae* | 1 | 0 |
|  | *Billaea triangulifera* | 0 | 10 |
|  | *Blondelia nigripes* | 0 | 5 |
|  | *Catharosia pygmaea* | 2 | 49 |
|  | *Chetogena obliquata* | 1 | 0 |
|  | *Cylindromyia interrupta* | 0 | 1 |
|  | *Dinera fuscata occidentalis* | 0 | 3 |
|  | *Drino atropivora* | 0 | 2 |
|  | *Epicampocera succincta* | 0 | 1 |
|  | *Eriothrix monticola* | 0 | 6 |
|  | *Eriothrix rufomaculatus* | 0 | 28 |
|  | *Estheria bohemani* | 0 | 21 |
|  | *Eumea mitis* | 0 | 1 |
|  | *Exorista paligera* | 0 | 2 |
|  | *Exorista rustica* | 0 | 1 |
|  | *Exorista rustica* group | 0 | 4 |
|  | *Exorista tubulosa* | 0 | 2 |
|  | *Hubneria affinis* | 0 | 5 |
|  | *Labigastera forcipata* | 0 | 6 |
|  | *Linnaemya comta* | 1 | 8 |
|  | *Loewia adjuncta* | 0 | 1 |
|  | *Loewia brevifrons* | 0 | 2 |
|  | *Loewia* cf. *adjuncta* | 0 | 1 |
|  | *Lydina aenea* | 2 | 1 |
|  | *Macquartia tenebricosa* | 2 | 4 |
|  | *Macquartia tessellum* | 1 | 0 |
|  | *Masistylum arcuatum* | 0 | 22 |
|  | *Meigenia dorsalis* | 4 | 0 |
|  | *Meigenia mutabilis* | 4 | 2 |
|  | *Meigenia mutabilis* group | 3 | 8 |
|  | *Nowickia ferox* | 0 | 3 |
|  | *Panzeria anthophila* | 0 | 1 |
|  | *Panzeria gemina* | 1 | 0 |
|  | *Panzeria intermedia* | 0 | 1 |
|  | *Panzeria* sp. | 0 | 1 |
|  | *Panzeria vivida* | 0 | 1 |
|  | *Peleteria rubescens* | 2 | 3 |
|  | *Periscepsia spathulata* | 0 | 1 |
|  | *Phryxe nemea* | 1 | 3 |
|  | *Phyllomya volvulus* | 1 | 1 |
|  | *Platymya fimbriata* | 0 | 2 |
|  | *Prosena siberita* | 1 | 1 |
|  | *Siphona pauciseta* | 0 | 1 |
|  | *Tachina fera* | 0 | 3 |
|  | *Tachina magnicornis* | 0 | 10 |
|  | *Thelaira leucozona* | 0 | 10 |
|  | *Triarthria setipennis* | 0 | 1 |
|  | *Voria ruralis* | 1 | 7 |

**Table S2** - List of plant species and average biomass estimated in 2021 and in 2023.

| **Species** | **Biomass** | |
| --- | --- | --- |
|  | **2021** | **2023** |
| *Abies alba* | 0.29 | 0.26 |
| *Acer pseudoplatanus* | 0.03 | 0.11 |
| *Achillea millefolium* agg. | 0.21 | 0.25 |
| *Aconitum lycoctonum* | 0.81 | 1.50 |
| *Aconitum napellus* | 0.05 | 0.12 |
| *Adenostyles alliariae* | 0.36 | 0.29 |
| *Adenostyles alpina* | 0.92 | 0.62 |
| *Aegopodium podagraria* | 0.20 | 0.32 |
| *Agropyron caninum* | 0.03 | 0 |
| *Agrostis tenuis* | 1.17 | 1.50 |
| *Ajuga pyramidalis* | 0 | 0.02 |
| *Ajuga reptans* | 0.41 | 0.10 |
| *Alchemilla* sp*.* | 0.88 | 0.66 |
| *Anemonoides trifolia* | 0.12 | 0.05 |
| *Anthoxanthum odoratum* | 0.35 | 0.39 |
| *Anthriscus sylvestris* | 0.07 | 0.02 |
| *Anthyllis vulneraria* | 0.03 | 0.02 |
| *Aposeris foetida* | 1.26 | 0.71 |
| *Aquilegia atrata* | 0.15 | 0.22 |
| *Arabis ciliata* | 0.02 | 0.02 |
| *Aruncus dioicus* | 0.07 | 0.20 |
| *Asplenium trichomanes* | 0 | 0.04 |
| *Athyrium filix-femina* | 1.20 | 0.97 |
| *Atropa belladonna* | 0.13 | 0.26 |
| *Barbarea bracteosa* | 0.05 | 0 |
| *Barbarea vulgaris* | 0.03 | 0 |
| *Bellis perennis* | 0.01 | 0 |
| *Bistorta officinalis* | 0.02 | 0.04 |
| *Brachypodium rupestre* | 0.57 | 1.79 |
| *Brachypodium sylvaticum* | 0.12 | 0.32 |
| *Briza media* | 0 | 0.02 |
| *Bromus benekenii* | 0 | 0.08 |
| *Calamagrostis arundinacea* | 1.40 | 0.93 |
| *Calamagrostis varia* | 0.16 | 0.62 |
| *Calamagrostis villosa* | 0.09 | 0.06 |
| *Campanula scheuchzeri* | 0.16 | 0.10 |
| *Cardamine bulbifera* | 0.05 | 0 |
| *Cardamine enneaphyllos* | 0.08 | 0 |
| *Cardamine hirsuta* | 0.11 | 0 |
| *Cardamine impatiens* | 0.12 | 0.17 |
| *Cardamine trifolia* | 0.27 | 0.10 |
| *Cardaminopsis halleri* | 0 | 0.04 |
| *Carduus defloratus* | 0.02 | 0.15 |
| *Carduus nutans* | 0.11 | 0.11 |
| *Carduus personata* | 0.25 | 0.12 |
| *Carex alba* | 3.25 | 2.28 |
| *Carex austroalpina* | 0.53 | 0.39 |
| *Carex digitata* | 0.19 | 0.44 |
| *Carex flacca* | 0.06 | 0.08 |
| *Carex leporina* | 0.23 | 0.37 |
| *Carex ornithopoda* | 1.03 | 0.57 |
| *Carex pairae* | 0.10 | 0.37 |
| *Carex pallescens* | 0.13 | 0.15 |
| *Carex panicea* | 0 | 0.02 |
| *Carex pilulifera* | 0 | 0.05 |
| *Carex sylvatica* | 0.20 | 0.43 |
| *Carum carvi* | 0.04 | 0 |
| *Centaurea nigrescens* | 0.03 | 0.04 |
| *Cerastium holosteoides* | 0.17 | 0.30 |
| *Chaerophyllum hirsutum* | 0.14 | 0.11 |
| *Chaerophyllum villarsii* | 0.01 | 0.03 |
| *Chrysosplenium alternifolium* | 0.03 | 0 |
| *Circaea alpina* | 0.05 | 0 |
| *Cirsium arvense* | 1.35 | 1.67 |
| *Cirsium eriophorum* | 0.07 | 0.08 |
| *Cirsium erisithales* | 1.25 | 1.25 |
| *Cirsium oleraceum* | 0.98 | 0.52 |
| *Cirsium vulgare* | 0.03 | 0.06 |
| *Clematis alpina* | 0.08 | 0.09 |
| *Clinopodium alpinum* | 0.01 | 0.04 |
| *Clinopodium grandiflorum* | 0.22 | 0.07 |
| *Crepis biennis* | 0 | 0.04 |
| *Cruciata glabra* | 1.33 | 1.07 |
| *Cruciata laevipes* | 0.17 | 0.63 |
| *Cyclamen purpurascens* | 0.11 | 0.10 |
| *Cynosurus cristatus* | 0 | 0.02 |
| *Cystopteris fragilis* | 0.06 | 0.06 |
| *Dactylis glomerata* | 0.54 | 0.62 |
| *Dactylorhiza fuchsii* | 0.50 | 0.56 |
| *Daphne mezereum* | 0.23 | 0.21 |
| *Deschampsia cespitosa* | 2.07 | 2.66 |
| *Doronicum austriacum* | 0.04 | 0 |
| *Dryopteris assimilis* | 0 | 0.02 |
| *Dryopteris carthusiana* | 0 | 0.03 |
| *Dryopteris filix-mas* | 0.25 | 0.29 |
| *Echium vulgare* | 0.05 | 0.02 |
| *Epilobium angustifolium* | 0 | 0.03 |
| *Epilobium montanum* | 0.79 | 0.57 |
| *Erigeron annuus* | 0.03 | 0.02 |
| *Eupatorium cannabinum* | 0.06 | 0.07 |
| *Euphorbia carniolica* | 1.08 | 0.58 |
| *Euphorbia cyparissias* | 0.31 | 0.44 |
| *Euphrasia* sp. | 0.01 | 0.02 |
| *Fagus sylvatica* | 0.70 | 0.40 |
| *Festuca altissima* | 0.02 | 0.03 |
| *Festuca arundinacea* | 0.03 | 0.03 |
| *Festuca nigrescens* | 1.77 | 1.71 |
| *Festuca rubra* | 0.10 | 0.14 |
| *Fragaria vesca* | 1.32 | 1.46 |
| *Fraxinus excelsior* | 0 | 0.11 |
| *Galeopsis pubescens* | 1.34 | 0.10 |
| *Galeopsis* sp. | 0.04 | 0 |
| *Galeopsis speciosa* | 0.39 | 0 |
| *Galeopsis tetrahit* | 1.04 | 0.26 |
| *Galium anisophyllum* | 0.23 | 0.22 |
| *Galium aparine* | 0 | 0.02 |
| *Galium centroniae* | 0.03 | 0.02 |
| *Galium mollugo* | 1.20 | 1.98 |
| *Gentiana asclepiadea* | 0.05 | 0.09 |
| *Gentiana cruciata* | 0 | 0.06 |
| *Geranium phaeum* | 0.06 | 0.12 |
| *Geranium purpureum* | 0.02 | 0.05 |
| *Geranium robertianum* | 0.06 | 0.09 |
| *Geranium sylvaticum* | 0.39 | 0.03 |
| *Geum rivale* | 0.61 | 0.64 |
| *Geum urbanum* | 0.02 | 0.04 |
| *Glechoma hederacea* | 0 | 0.05 |
| *Gnaphalium sylvaticum* | 0.02 | 0.03 |
| *Gymnocarpium dryopteris* | 0.32 | 0.16 |
| *Gymnocarpium robertianum* | 0.01 | 0.02 |
| *Helleborus viridis* | 0.19 | 0.17 |
| *Heracleum sphondylium* | 0.06 | 0.03 |
| *Hieracium murorum* | 1.71 | 2.13 |
| *Homogyne alpina* | 0.25 | 0.06 |
| *Hypericum maculatum* | 0.96 | 1.37 |
| *Hypericum perforatum* | 0.08 | 0.23 |
| *Impatiens glandulifera* | 0 | 0.02 |
| *Jacobaea alpina* | 0.24 | 0.18 |
| *Juncus articulatus* | 0 | 0.04 |
| *Juncus effusus* | 0.06 | 0.07 |
| *Knautia drymeia* | 0.12 | 0.14 |
| *Koeleria pyramidata* | 0 | 0.04 |
| *Lamium galeobdolon* | 0.89 | 0.82 |
| *Lamium orvala* | 0 | 0.04 |
| *Lapsana communis* | 0 | 0.06 |
| *Lathyrus pratensis* | 0.29 | 0.51 |
| *Leontodon hispidus* | 0.03 | 0.09 |
| *Leucanthemum vulgare* | 1.10 | 0.79 |
| *Linaria vulgaris* | 0.02 | 0.04 |
| *Lonicera alpigena* | 0.08 | 0.20 |
| *Lonicera nigra* | 2.85 | 3.72 |
| *Lonicera xylosteum* | 0.44 | 0.43 |
| *Lotus corniculatus* | 0.24 | 0.39 |
| *Luzula luzulina* | 0.53 | 0.33 |
| *Luzula luzuloides* | 0.06 | 0 |
| *Luzula multiflora* | 0.13 | 0.20 |
| *Luzula nivea* | 2.93 | 3.41 |
| *Luzula pilosa* | 0.20 | 0.28 |
| *Luzula sylvatica* | 0.53 | 0.17 |
| *Maianthemum bifolium* | 0.27 | 0.16 |
| *Medicago lupulina* | 0.12 | 0.14 |
| *Melampyrum sylvaticum* | 0.48 | 0.30 |
| *Melica nutans* | 0.49 | 0.41 |
| *Melilotus officinalis* | 0 | 0.02 |
| *Mentha longifolia* | 0 | 0.07 |
| *Mercurialis perennis* | 0.13 | 0.07 |
| *Moehringia muscosa* | 0.14 | 0.06 |
| *Mycelis muralis* | 0.28 | 0.36 |
| *Myosotis* sp. | 1.00 | 0.23 |
| *Myrrhis odorata* | 0.03 | 0.28 |
| *Oxalis acetosella* | 0.18 | 0 |
| *Paris quadrifolia* | 0.06 | 0.04 |
| *Pedicularis tuberosa* | 0.03 | 0.05 |
| *Pedicularis verticillata* | 0.02 | 0 |
| *Petasites albus* | 2.67 | 1.29 |
| *Petasites paradoxus* | 0.27 | 0 |
| *Phleum pratense* | 0.04 | 0 |
| *Phleum rhaeticum* | 0.23 | 0.27 |
| *Phyteuma betonicifolium* | 0.07 | 0.06 |
| *Phyteuma orbiculare* | 0 | 0.02 |
| *Phyteuma ovatum* | 0 | 0.04 |
| *Phyteuma spicatum* | 0.44 | 0.47 |
| *Picea abies* | 0.24 | 0.59 |
| *Picris hieracioides* | 0.04 | 0.02 |
| *Pimpinella saxifraga* | 0.01 | 0.04 |
| *Plantago lanceolata* | 0.02 | 0.02 |
| *Plantago major* | 0.14 | 0.08 |
| *Plantago media* | 0.04 | 0.04 |
| *Poa alpina* | 0.05 | 0 |
| *Poa annua* | 0.07 | 0 |
| *Poa nemoralis* | 0.09 | 0.36 |
| *Poa pratensis* | 0.51 | 0.49 |
| *Poa trivialis* | 1.55 | 1.19 |
| *Polygala alpestris* | 0 | 0.02 |
| *Polygonatum verticillatum* | 1.20 | 0.98 |
| *Polygonum aviculare* | 0.01 | 0 |
| *Populus tremula* | 0 | 0.02 |
| *Potentilla aurea* | 0.04 | 0.02 |
| *Potentilla crantzii* | 0.04 | 0 |
| *Potentilla erecta* | 0.17 | 0.30 |
| *Prenanthes purpurea* | 1.43 | 0.91 |
| *Prunella vulgaris* | 0.27 | 0.64 |
| *Pulmonaria officinalis* | 0.02 | 0.02 |
| *Ranunculus acris* | 0.51 | 0.27 |
| *Ranunculus bulbosus* | 0 | 0.02 |
| *Ranunculus* gr. *montanus* | 0.02 | 0.06 |
| *Ranunculus nemorosus* | 0 | 0.02 |
| *Ranunculus platanifolius* | 0.44 | 0.25 |
| *Ranunculus repens* | 0.48 | 0.35 |
| *Rhinanthus freynii* | 0.15 | 0.20 |
| *Ribes uva-crispa* | 0 | 0.05 |
| *Rosa pendulina* | 0.19 | 0.25 |
| *Rosa* sp. | 0.26 | 0.19 |
| *Rubus idaeus* | 7.60 | 10.47 |
| *Rubus saxatilis* | 0.02 | 0.22 |
| *Rubus* sp. | 2.08 | 2.12 |
| *Rumex acetosa* | 0.08 | 0.16 |
| *Rumex acetosella* | 0 | 0.17 |
| *Rumex obtusifolius* | 0.09 | 0.18 |
| *Salix appendiculata* | 0.41 | 1.13 |
| *Sambucus nigra* | 0 | 0.08 |
| *Sambucus racemosa* | 1.24 | 1.40 |
| *Saxifraga rotundifolia* | 0.14 | 0.14 |
| *Scrophularia nodosa* | 0.72 | 0.48 |
| *Senecio cacaliaster* | 1.95 | 1.16 |
| *Senecio inaequidens* | 0.07 | 0.18 |
| *Senecio nemorensis* | 0.52 | 0.55 |
| *Senecio rupestris* | 0.06 | 0.14 |
| *Sesleria caerulea* | 0.02 | 0 |
| *Silene dioica* | 0.18 | 0.21 |
| *Solanum dulcamara* | 0.22 | 0.20 |
| *Solidago virgaurea* | 0.52 | 0.34 |
| *Sonchus* sp. | 0.05 | 0 |
| *Sorbus aucuparia* | 0.55 | 0.99 |
| *Stachys alpina* | 0.06 | 0.07 |
| *Stachys sylvatica* | 0.09 | 0.26 |
| *Stellaria graminea* | 0.12 | 0.18 |
| *Stellaria media* | 0.06 | 0.02 |
| *Stellaria nemorum* | 0.16 | 0.08 |
| *Symphytum tuberosum/bulbosum* | 0.10 | 0.18 |
| *Taraxacum* sp. | 0.46 | 0.47 |
| *Telekia speciosa* | 0.58 | 1.23 |
| *Thlaspi alliaceum* | 0.02 | 0 |
| *Thymus polytrichus* | 0.04 | 0.04 |
| *Trifolium hybridum* | 0.13 | 0.02 |
| *Trifolium pratensis* | 0.75 | 0.79 |
| *Trifolium repens* | 0.47 | 0.44 |
| *Trollius europaeus* | 0.45 | 0.20 |
| *Tussilago farfara* | 2.09 | 1.79 |
| *Urtica dioica* | 2.37 | 2.02 |
| *Vaccinium myrtillus* | 1.67 | 0.95 |
| *Vaccinium vitis-idaea* | 0.07 | 0.11 |
| *Valeriana officinalis* | 0.12 | 0.28 |
| *Valeriana tripteris* | 0.62 | 0.54 |
| *Veratrum album* | 0.08 | 0.05 |
| *Verbascum alpinum* | 0.05 | 0 |
| *Verbascum nigrum* | 0.09 | 0.04 |
| *Verbascum thapsus* | 0.59 | 0.10 |
| *Veronica chamaedrys* | 0.69 | 0.70 |
| *Veronica officinalis* | 1.54 | 1.74 |
| *Veronica serpyllifolia* | 0.04 | 0 |
| *Veronica urticifolia* | 0.61 | 0.67 |
| *Vicia cracca* | 0 | 0.08 |
| *Vicia sativa* | 0.01 | 0.02 |
| *Vicia sylvatica* | 0 | 0.04 |
| *Viola biflora* | 0.17 | 0.02 |
| *Viola* sp. | 0.28 | 0.16 |

**Figure S1** – Diagnostic plots of models’ residuals’ normality and homogeneity of variance. Models are fitted with *D*_%diff_, *D*_turn_, and *D*_gain_ as response variables and the interaction between taxon and elevation and between taxon and windthrow extent as explanatory variables.

***D*_%diff_**


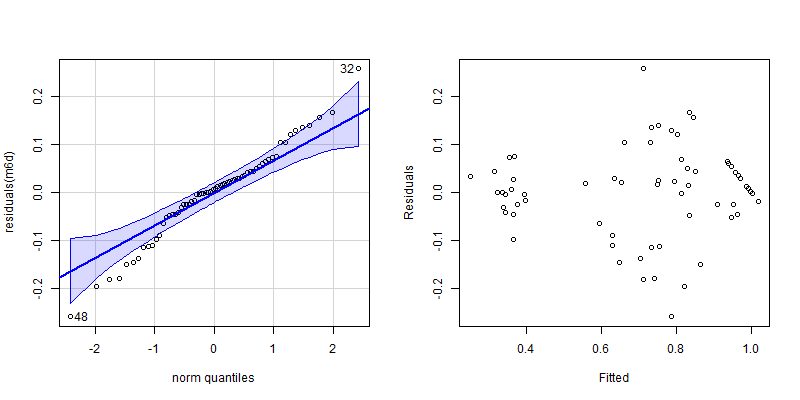


***D*_turn_**

**_
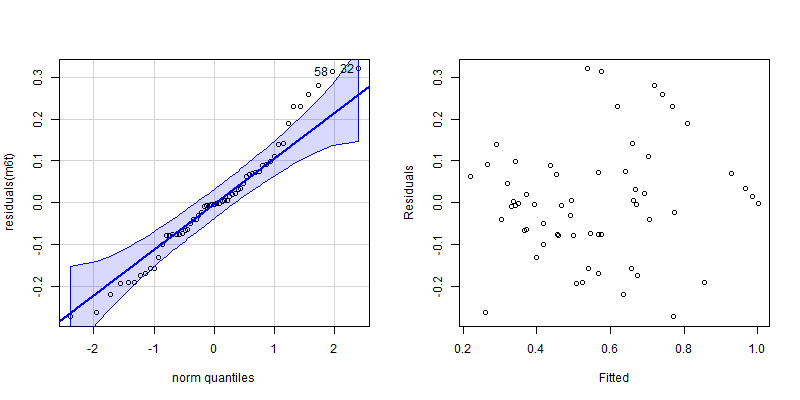
_**

***D*_gain_**

**_
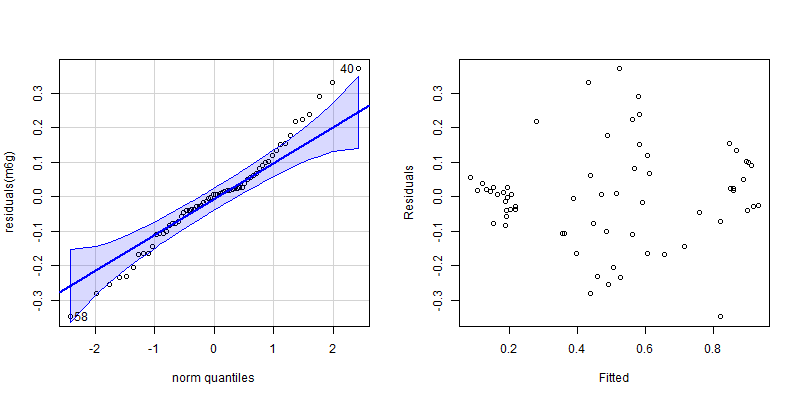
_**
